# The interplay between the formation of Chinese cordyceps and the characteristics of soil properties and microbial network

**DOI:** 10.1128/spectrum.03277-24

**Published:** 2025-05-30

**Authors:** Qinghe Wang, Yapei Wang, Ting Li, Xiuwen Bao, Liying He, Lin Liu, Sijing Liu, Jing Bai, Han Zhang, Shuqi Niu, Jinlin Guo

**Affiliations:** 1 School of Pharmacy, Chengdu University of Traditional Chinese Medicine 658555, Chengdu, Sichuan, China; 2 College of Medical Technology, Chengdu University of Traditional Chinese Medicine 118385, Chengdu, China; Fujian Agriculture and Forestry University, Fuzhou City, Fujian, China

**Keywords:** *Ophiocordyceps sinensis*, bacterial community, formation mechanism, soil enzyme activity, functional profiles

## Abstract

Understanding the interplay between soil properties and microbial communities is crucial for elucidating the ecological mechanisms driving Chinese cordyceps (CC) formation. However, few studies have explored the dynamic changes in soil properties and microbial communities at different stages of CC formation. This study presents a comprehensive investigation of soil properties and bacterial communities across the developmental stages of CC. Our results showed that the mummified host larvae-rearing soil (MS) stage exhibited the most pronounced fluctuations in critical soil parameters, including soil pH, organic matter, total phosphorus, available phosphorus, and potassium levels, suggesting heightened nutrient mobilization to support CC growth. Enzyme activities analysis further revealed that CC growth notably altered the activities of acid phosphatase and dehydrogenase in the soil during the MS stage. 16S rRNA high-throughput sequencing analysis revealed that as CC developed, both bacterial richness and the abundance of specific bacterial genera, particularly *Dyella* and *Pseudomonas*, increased. Furthermore, microbial network analysis indicated that the network became most complex and unstable during the MS stage. The formation of CC was closely linked to changes in soil properties and the characteristics of the bacterial network. Additionally, functional prediction analysis revealed a significant upregulation of chitinolytic function during the MS stage, highlighting its ecological function in CC development. These findings provide foundational data for understanding the mechanisms underlying CC formation and for optimizing artificial CC production to achieve higher yield and better quality.

**IMPORTANCE:**

This study elucidates the interactions between soil properties and microbial communities in regulating the growth and development of CC. Key findings include: (i) dynamic fluctuations in soil physicochemical properties during CC formation, with significant nutrient depletion during the MS stage; (ii) distinct microbial community succession, showing the highest network complexity but lowest stability during the MS stage; (iii) functional prediction revealed enhanced chemoheterotrophy and chitinolysis in MS, indicating microbial adaptive regulation. These results provide a theoretical foundation for deciphering the molecular mechanisms of CC formation and optimizing its production.

## INTRODUCTION

Chinese cordyceps (CC) is a parasitic fungus endemic to the Qinghai-Tibet Plateau, characterized by its unique life cycle that involves the fungus infecting the host larvae (*Thitarodes*, Hepialidae, Lepidoptera). The fungus *Ophiocordyceps sinensis* (Berk.) attaches to the host, eventually consuming it and emerging as a fungal structure integrated with larval remnants (1–3). Studies have shown that the interaction between CC and its host larvae significantly impacts local ecosystems. By affecting host larvae populations, the fungus alters the broader ecological balance, including plant communities and soil health (4–6). Its medicinal properties have been recognized for centuries, particularly in East Asia, where it is used for its purported benefits in enhancing vitality and treating various ailments. The main components of CC include polysaccharides, proteins, amino acids, and nucleosides used in immunomodulatory, anti-inflammatory, antioxidant, and anti-tumor applications (7–9). The medicinal properties further confer considerable economic value in CC, with the industry estimated at 30–50 billion yuan (10). However, its high economic demand has led to over-exploitation, causing a decline in wild populations. To mitigate environmental damage and optimize its medicinal potential, significant progress has been made in CC artificial cultivation research (11, 12). By 2024, the artificial cultivation of CC had reached a certain scale, but challenges such as low infection rates and slow development persisted. Addressing these issues requires a deeper understanding of the interaction mechanisms between CC and host larvae.

CC is exclusively found at altitudes above 3,000 m, predominantly across the Qinghai-Tibet Plateau (13). Its distribution spans several countries, including China, Bhutan, India, and Nepal, where the specific climatic and soil conditions of these high-altitude regions facilitate its growth (14–16). CC exhibits a highly specialized parasitic relationship with the host larvae. After spore release, CC spores deposit in the soil and infect the host larvae, gradually replacing the larval tissue with fungal mycelium. The parasitism not only facilitates the fungus’s growth but also influences the ecological dynamics of the host’s population, demonstrating a complex interplay between the CC and its environment (17). Soil plays a crucial role in both field growth and artificial cultivation of CC. It serves not only as the habitat for the host larvae but also forms a protective matrix with surrounding hyphae, providing essential nutrients and safeguarding the developing CC. There is a lack of research on soil changes during CC formation, particularly regarding alterations in soil properties and microbial communities.

Previous studies have identified significant variations in bacterial communities across different occurrence sites of CC. Notably, sites with higher occurrences show greater bacterial diversity and evenness (4). Additionally, soil properties and microbial characteristics have been implicated in the prevalence of CC (18). Research on *O. highlandensis*, a closely related species, has revealed considerable fluctuations in bacterial community composition and function during the development of its fruiting body .This suggests that *O. highlandensis* may recruit distinct bacterial populations to support growth and facilitate rapid infection throughout its lifecycle (19). However, few studies have examined the soil properties and microbial dynamics associated with different developmental stages of CC. Therefore, this study investigates changes in soil properties and microbial communities throughout the formation of CC, aiming to provide foundational data for the artificial cultivation of high-quality and high-yield CC.

This study investigated the changes in soil physicochemical properties, enzyme activities, and microbial diversity during the formation of CC. Specifically, the research examines if and how CC affects soil properties, microbial diversity, and network characterization. The study addresses the following questions: (i) Does CC formation affect soil properties? (ii) Does it alter soil microbial communities? (iii) How do the processes involved in CC formation influence the complexity and stability of soil microbial networks? (iv) Are changes in soil microbial networks associated with soil properties, and if so, what are the primary drivers of these changes? This study aims to inform strategies for sustainable management and conservation, ensuring the long-term viability of both the fungus and the ecosystems it supports.

## MATERIALS AND METHODS

### Soil sample collection

The artificial cultivation of CC involves rearing the host larvae (*Thitarodes xiaojinensis*), infecting them with *O. sinensis*, and then planting the mummified host larvae in the soil to induce CC formation. Soil samples were collected to monitor changes in microbial communities and soil properties associated with this process. For this study, various soil samples were collected from the laboratory (30°41′N, 103°47′E). Thses samples were categorized into sterilized soil (SS) for baseline comparisons, host larvae-rearing soil (HS, pre-mummification) to assess pre-infection conditions, mummified host larvae-rearing soil (MS) to evaluate the impact of mummification, and pellicle soil (PS, comprising soil particles and mycelium attached to the CC) to understand the interactions between mycelium and soil. Soil collection was performed using a sterile shovel to excavate solid CC soil and a sterile spoon to gather samples, ensuring minimal contamination. The collection was done with care to maintain the integrity of the soil layers and microbial communities. Soil slices, 0–15 cm deep and 10 cm wide, were extracted. Eight plots were sampled as biological replicates. Each soil sample was divided into two parts: one was immediately stored at −80°C for DNA extraction, while the other was stored at 4°C for measurement of soil enzyme activities.

### Analysis of soil physicochemical properties and enzyme activities

The soil physicochemical properties analyzed in this study included pH, organic matter (OM), total nitrogen (TN), total phosphorus (TP), total potassium (TK), alkaline-hydrolyzed nitrogen (AN), available phosphorus (AP), and available potassium (AK). The methods for these analyses were based on procedures outlined in *Soil Analysis in Agricultural Chemistry* (20). Soil pH was measured using a calibrated pH meter, following the manufacturer’s instructions. OM content was determined using the Walkley-Black method. TN, TP, and TK were quantified though the Kjeldahl digestion method, the molybdenum antimony anti-colorimetry method, and flame atomic absorption spectrophotometry, respectively. AN was evaluated by mixing the soil with potassium hydroxide, heating the mixture to release nitrogen compounds, and neutralizing before distillation; ammonium ions (NH_4_^+^) were then titrated to estimate nitrogen availability. AP and AK were determined using the molybdenum antimony spectrophotometric method and flame atomic absorption spectrophotometry, respectively.

Additionally, we measured six enzymes associated with soil health: acid phosphatase activity (ACP), invertase activity (SC), urease activity (UE), dehydrogenase activity (DHA), polyphenol oxidase activity (PPO), and peroxidase activity (POD). These measurements were conducted on air-dried soil that had passed through a 50-mesh sieve. All enzyme activities were measured using a reagent kit provided by Suzhou Mengxi Biopharmaceutical Co., Ltd.

### DNA extraction, amplification, sequencing, and sequence data analysis

Total genomic DNA was extracted from samples using the TGuide S96 Magnetic Soil/Stool DNA Kit (Tiangen Biotech, Beijing, China). DNA concentration and purity were assessed using a NanoDrop 2000 UV-Vis spectrophotometer (Thermo Scientific, Wilmington, USA). The full-length 16S rRNA gene was amplified using primer pairs 27F (AGRGTTTGATYNTGGCTCAG) and 1492R (TASGGHTACCTTGTTASGACTT) (21). Each PCR reaction had a total volume of 30 µL, which included 15 µL of KOD One PCR Master Mix (Beijing Bailingke Biotechnology, China), 2.25 µL (at a concentration of 0.3 µM) primer pairs, 3 µL template DNA (approximately 200 ng/µL), and 9.75 µL of PCR-grade water. To ensure accuracy, each sample was amplified in triplicate using the following thermal cycling program: an initial denaturation at 95°C for 2 min, followed by 22 cycles consisting of denaturation at 98°C for 10 s, annealing at 55°C for 30 s, and extension at 72°C for 1 min 30 s. The process concluded with a final extension at 72°C for 2 min. The resulting PCR products were analyzed using agarose gel electrophoresis at a concentration of 1.8%. The electrophoresis was carried out under the following conditions: a voltage of 120 V for approximately 40–45 min, with a 250 bp marker for reference. The target band, which corresponded to a size of about 1,500 bp, appeared distinct and well-defined, confirming successful amplification and proper positioning on the gel. PCR amplicons were purified using VAHTS DNA Clean Beads (Vazyme, Nanjing, China) and quantified with the Qubit dsDNA HS Assay Kit and Qubit 3.0 Fluorometer (Invitrogen, Thermo Fisher Scientific, USA). After quantification, amplicons were pooled in equal amounts. SMRTbell libraries were prepared from the amplified DNA using the SMRTbell Express Template Prep Kit 2.0 (Pacific Biosciences) according to the manufacturer’s instructions. The purified SMRTbell libraries were sequenced on a PacBio Sequel II platform (Beijing Biomarker Technologies Co., Ltd.) using the Sequel II Binding Kit 2.0.

Bioinformatics analysis was conducted using the BMK Cloud platform (http://www.biocloud.net/). Raw sequencing reads were filtered and demultiplexed using SMRT Link software (v8.0) with parameters set at minimum passes ≥ 5 and minimum predicted accuracy ≥ 0.9 to generate circular consensus sequencing (CCS) reads. The lima tool (version 1.7.0) was used to assign CCS sequences to their respective samples based on barcodes. CCS reads without primers and those outside the length range of 1,200–1,650 bp were discarded using Cutadapt (22) (v2.7) for quality control. Chimera sequences were detected and removed using the UCHIME algorithm (v8.1) to obtain clean reads. Sequences with similarity > 97% were clustered into operational taxonomic units (OTUs) using USEARCH (23) (v10.0), and OTUs with counts less than 2 across all samples were filtered out.

### Statistical analysis

Soil physicochemical properties and enzyme activities were analyzed using one-way ANOVA with Tukey’s HSD test for analysis. Graphs were generated using GraphPad Prism (v9.5.0).

Bioinformatics analysis of soil bacteria was conducted based on OTUs using the BMK Cloud (www.biocloud.net). One-way ANOVA with Tukey’s HSD test was used to analyze the Alpha diversity indices including the ACE, Chao1, Shannon, Simpson, and PD whole tree indices, across different soil stages. Nonmetric multidimensional scaling (NMDS) was performed using both weighted and unweighted Unifrac distances, and differences in community composition were assessed through permutational multivariate analysis of variance. Additionally, the relative abundances of the top 10 phyla and genera were compared using one-way ANOVA with Tukey’s HSD test. All the OTUs data were normalized (to make the different features comparable), then the data were converted into log_2_ (FC) and −log_10_ (*P* value) formats, and then the data were imported into OmicStudio tools (https://www.omicstudio.cn/tool) for visualization. ChiPlot (https://www.chiplot.online/) was used to perform Pearson correlation analysis between bacterial microbial communities and soil physicochemical factors. The Mantel test was applied to assess the significance of these correlations. Furthermore, the FAPROTAX pipeline was used to predict the putative functions of the bacterial community and estimate the impact of CC’s formation on the properties of soil microbial functions.

### Microbial co-occurrence network construction, network stability, and identification of keystone species

A molecular ecological network was constructed using OTUs relative abundance data. Microbial co-occurrence patterns were analyzed using iNAP, a comprehensive network analysis pipeline for microbiome studies (24). Initially, at least seven out of eight biological replicates were retained based on criteria for data quality and completeness criteria. Next, the Spearman correlation coefficient |*r*| > 0.6 and *P* < 0.001 between the filtered OTUs was calculated. The *P* values have been adjusted for multiple testing using the Benjamini-Hochberg’s FDR method. Then a similarity matrix was obtained. Applying random matrix theory, the adjacent correlation coefficient method determined the appropriate cut-off value for network construction. Subsequently, a network was generated using this cut-off value, with a correlation matrix and *P* value matrix to define node and link attributes. To estimate the complexity of the soil networks, we calculated various network characteristics, including total number of nodes, number of links, number of modules, modularity, *R*^2^ of power law, average degree (avgK), average path distance GD, harmonic geodesic distance (HD), number of keystone species, average clustering coefficient (average CC), and Connectedness (Con). Network stability was assessed by randomly removing nodes and evaluating the impact on global efficiency using iNAP (25, 26). Finally, the network was visualized using Gephi (v0.9.2).

## RESULTS

### Variation in soil physicochemical properties and enzyme activities

**Fig 1:**
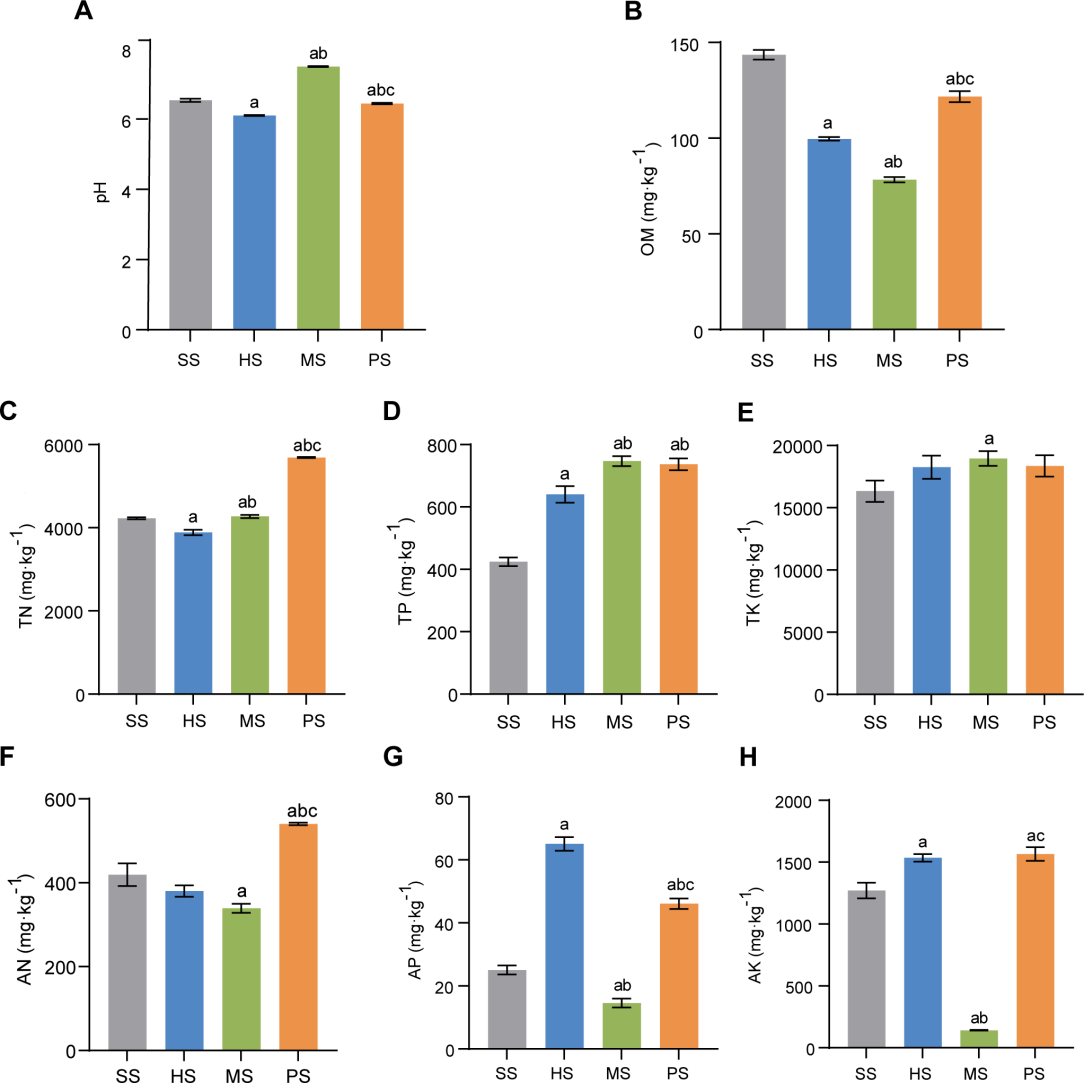
Changes in soil physicochemical properties across different developmental stages. (**A**) pH. (**B**) Organic matter (OM). (**C**) Total nitrogen (TN). (**D**) Total phosphorus (TP). (**E**) Total potassium (TK). (**F**) Alkaline-hydrolyzed nitrogen (AN). (**G**) Available phosphorus (AP). (**H**) Available potassium (AK). Lowercase letters indicate significant differences (*P* < 0.05): a vs SS; b vs HS; and c vs MS.

Different stages of CC significantly affected the soil physicochemical properties (Fig. 1). The soils in SS, HS, and PS stages were acidic, while the MS stage soil was alkaline, as indicated by the pH value (Fig. 1A). OM content decreased by 30.61%, 45.45%, and 15.21% in the HS, MS, and PS stages, indicating significant consumption of OM during HS and MS stages. (Fig. 1B). Compared with SS, the TN content initially decreased in HS but increased at MS and PS stages, with TN content in PS stage increasing by 34.53% (Fig. 1C). TP content increased by 50.90%, 76.04%, and 73.61% in HS, MS, and PS stages, respectively (Fig. 1D). Similar trends were observed for TK content (Fig. 1E). AN content significantly decreased during the HS and MS stages but increased significantly by 28.96% in the PS stage (Fig. 1F). This suggests N consumption in the HS and MS stages, with a subsequent increase in availability in the PS stage. Compared with SS, AP and AK contents increased in the HS stage, decreased sharply in the MS stage, but rebounded in the PS stage (Fig. 1G and H). Overall, these fluctuations reflect high nutrient demands during CC growth.

**Fig 2:**
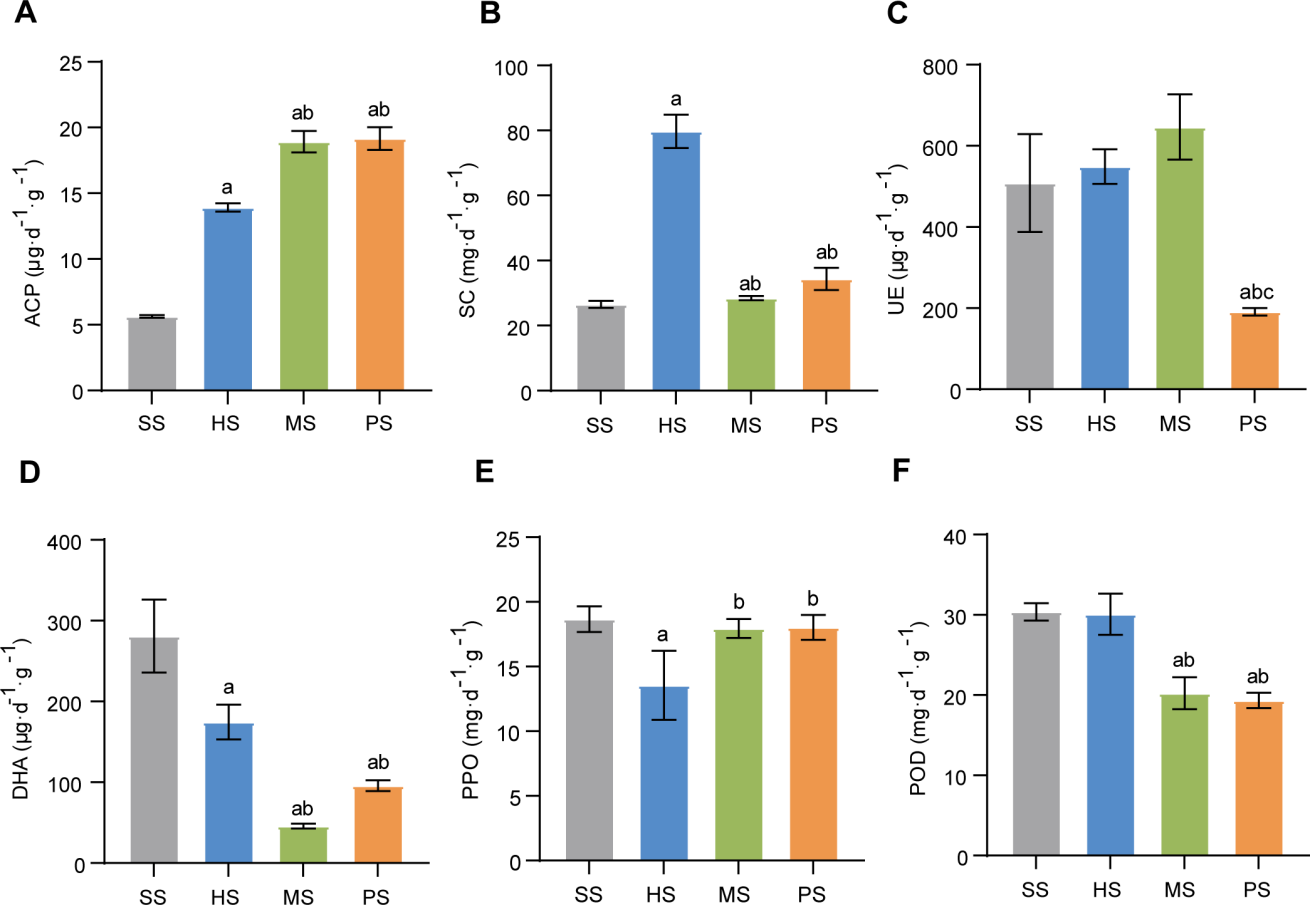
Changes in soil enzyme activities at different developmental stages. (**A**) Acid phosphatase (ACP). (**B**) Invertase (SC). (**C**) Urease (UE). (**D**) Dehydrogenase (DHA). (**E**) Polyphenol oxidase (PPO). (**F**) Peroxidase (POD). Lowercase letters indicate significant differences (*P* < 0.05) between stages: a vs SS; b vs HS; and c vs MS.

During CC formation, soil enzyme activities exhibited significant variations (Fig. 2). The ACP content increased progressively across SS, HS, MS, and PS stages, with a 239.71% rise in PS stage compared to SS stage (Fig. 2A). In contrast, SC activity peaked at the HS stage (Fig. 2B), suggesting enhanced soil fertility during host larval feeding. UE activity rose gradually from HS to MS stage but declined sharply in PS stage (Fig. 2C), indicating microbial community shifts post-stroma formation. Conversely, DHA activity gradually decreased from SS to MS stage, reaching its lowest at the MS stage before recovering in the PS stage (Fig. 2D), implying DHA depletion during CC cultivation and subsequent reactivation after stroma maturation. The PPO activity in HS stage was significantly lower than that in SS, MS, and PS stages (Fig. 2E), reflecting suppression during larval development. However, the activity of POD was markedly reduced in MS and PS compared to SS and HS stages (Fig. 2F).

### Microbial diversity in soil across different CC stages

**Fig 3:**
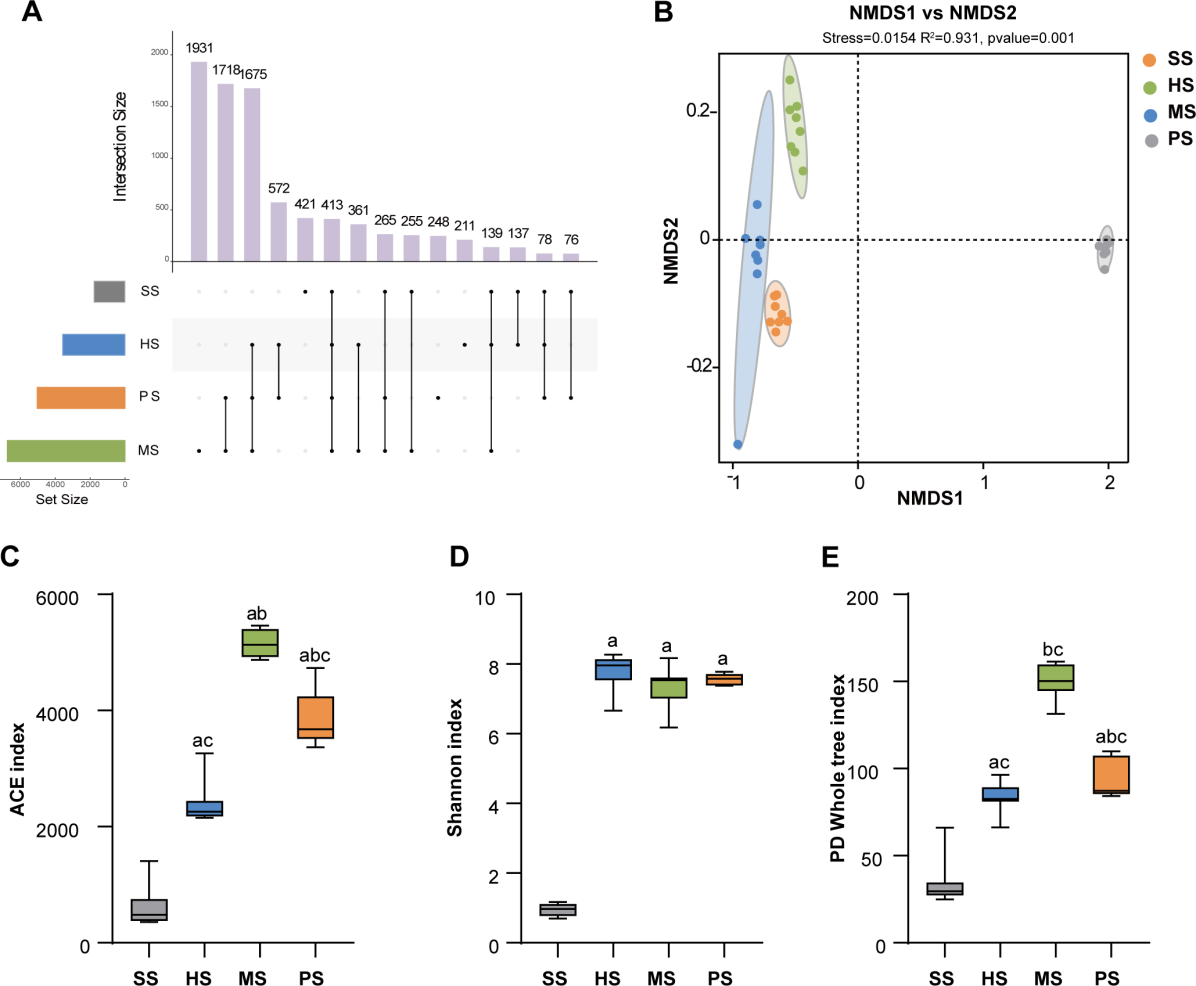
Soil microbial diversity across developmental stages. (**A**) Upset Venn diagram of obtained OTUs. (**B**) NMDS analysis of the microbial community in SS, HS, MS, and PS using weighted UniFrac distance metric. (**C**) ACE index. (**D**) Shannon index. (**E**) PD whole tree index across developmental stages. Lowercase letters indicate significant differences (*P* < 0.05): a vs SS; b vs HS; and c vs MS.

A total of 1,717,389 reads were obtained from 32 samples across four soil stages, resulting in 35 phyla, 79 classes, 232 orders, 449 families, 1,047 genera, 2,690 species, and 8,500 OTUs. The MS stage had the highest OTU richness, accounting for 79.49%. All samples shared 413 OTUs, representing 4.86% of the total, with unique OTUs ranging from 211 to 1,931 per stage, constituting less than 22.72% of the total OTUs (Fig. 3A). Significant compositional variations were observed among stages, with the MS stage exhibiting the highest number of unique OTUs, indicating a substantial microbial presence at this stage.

Differences in microbial community structures among soil stages were evaluated using NMDS. The samples exhibited high similarity, indicating high reliability (Fig. 3B and Fig. S1A). The analysis of HS, MS and PS stages further confirmed robust clustering (stress < 0.1; Fig. S1B and C). Soil microbial composition varied substantially across stages. To assess microbial diversity in each sample, we calculated various diversity indices, including rarefaction curves, Chao 1, Simpson, ACE, Shannon, and PD Whole tree indices. These metrics collectively provided insights into the richness, diversity, and evenness (Fig. 3C through E and Fig. S1D through F). A rarefaction curve plateaued beyond 40,000 reads (Fig. S1D), confirming sequencing depth sufficiency. Reaching this saturation point suggested that the results were reliable and accurately reflected the microbial diversity present in the samples. The MS stage exhibited the highest species richness (ACE/Chao indices; *P* < 0.05, partial *η^2^*= 0.96) compared to other stages (Fig. 3C and S1E). Species diversity in HS, MS, and PS stages was significantly greater than in SS stage (Simpson/Shannon indices, *P <* 0.05, partial *η^2^* = 0.98; *P <* 0.05, partial *η^2^* = 0.99 [approximated as 1.00], Fig. 3D and Fig. S1F). Similarly, community diversity was significantly greater in HS, MS, and PS stages compared to SS stage (*P* < 0.05, partial *η^2^* = 0.94, Fig. 3E).

To assess changes in soil microbial composition during CC culturing, we analyzed the phylum and genus profiles across SS, HS, MS, and PS stages, focusing on the top 10 most abundant taxa (Fig. S2). Compared to SS stage, the relative abundances of Proteobacteria, Bacteroidota, and Verrucomicrobiota were significantly higher in HS, MS, and PS stages (Fig. S2A). Firmicutes showed a notable increase in MS compared to HS and PS. At the genus level, *Chitinophaga*, *Pedobacter*, and *Rhodanobacter* exhibited enrichment in HS, MS, and PS stages compared to SS stage. *Dyella* exhibited enrichment in HS, and MS stages compared to SS stage. *Pseudomonas*, *Mucilaginibacter*, and *Pseudoxanthomonas* exhibited enrichment in HS, and PS stages compared to SS stage. *Chitinophaga* reached its peak abundance in MS but remained scarce in HS and PS stages (Fig. S2B).

### Association of significantly enriched OTUs with CC stages in soil

**Fig 4:**
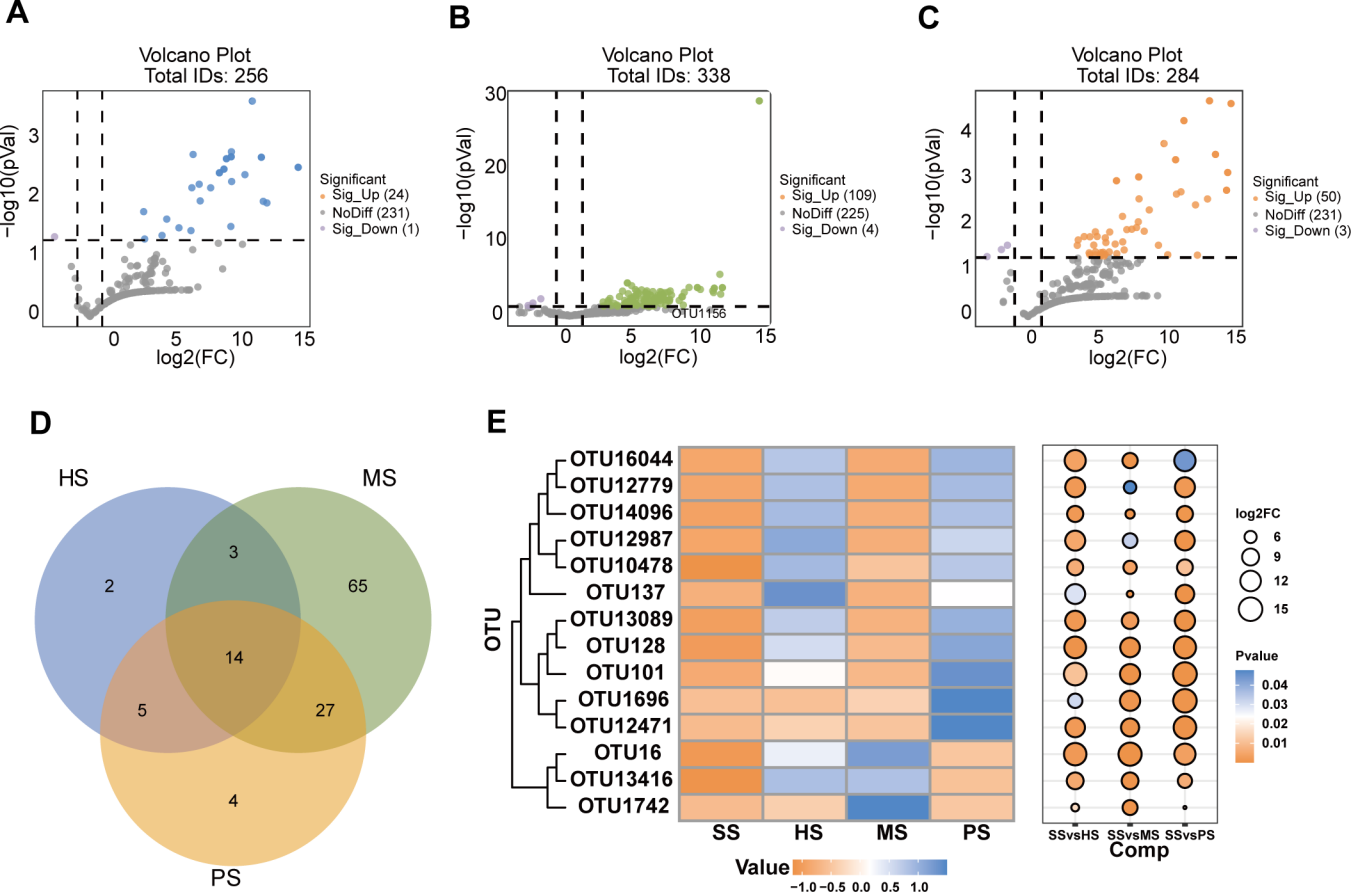
Differential OTU abundance patterns across developmental stages. Enrichment and depletion of the OTUs for HS (**A**), MS (**B**), and PS (**C**) compared with SS. (**D**) Counts of stage-specific enriched OTUs relative to SS. (**E**) Heatmap bubble plot displaying commonly enriched OTUs with differential abundance compared to SS.

To identify OTUs associated with community divergence across soil stages, we conducted an abundance analysis. Compared to SS stage, results revealed a substantial increase in enriched OTUs at the HS stage (24 enriched vs 1 depleted). This trend intensified during the MS stage, with 109 enriched OTUs versus only 4 depleted OTUs. Notably, the PS stage showed reduced differential OTUs (50 enriched vs 3 depleted), indicating peak microbial enrichment during the MS stage of CC cultivation (Fig. 4A through C).

Notable overlaps were observed among differentially enriched OTUs across soil stages (Fig. 4D). Fourteen OTUs, primarily comprising Proteobacteria, Bacteroidota, Actinobacteriota, and Firmicutes, were differentially enriched in all soil stages compared to SS (Table S1). These OTUs exhibited distinct distribution patterns. In particular, *Pedobacter* (OTU137), *Chitinophaga* (OTU101), and *Verticillia* (OTU12987) were predominant in HS stage. *Dyella* (OTU16), *Ramlibacter* (OTU13416), *Paenibacillus* (OTU1742), and *Pseudomonas* (OTU16044) were relatively abundant in MS stage. At the same time, *Chitinophaga* (OTU101), *Leuconostoc* (OTU1696), and *Pedobacter* (OTU12471) were more abundant in PS stage (Fig. 4E). The MS stage showed the highest microbial abundance, with significant genus-level changes (e.g., *Dyella* and *Pseudomonas*).

### Microbial network complexity and stability

**Fig 5:**
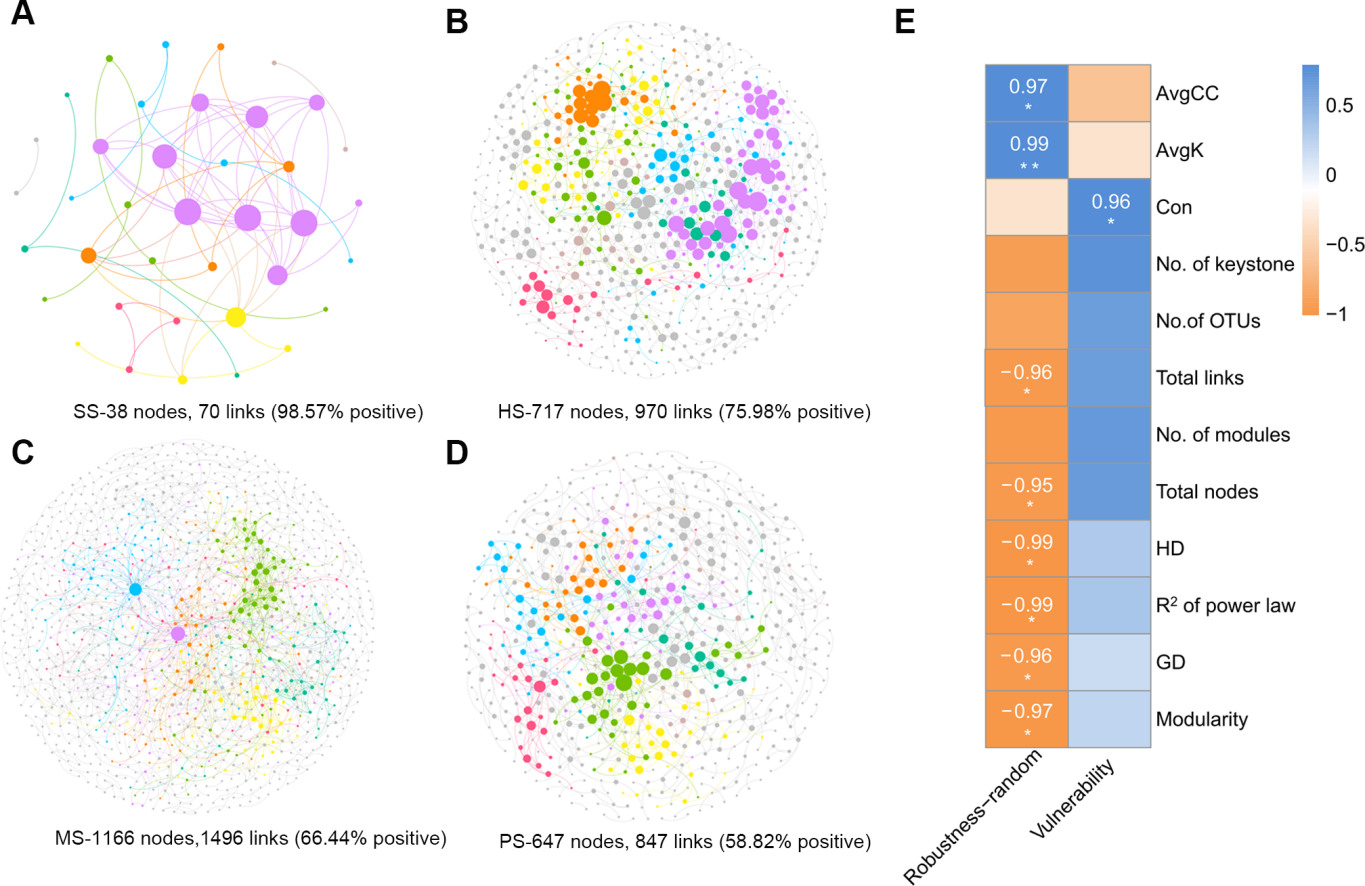
Bacterial co-occurrence network analysis. Microbial co-occurrence patterns in the SS (**A**), HS (**B**), MS (**C**), and PS (**D**). Nodes represent OTUs, with node size proportional to degree, indicating centrality. Nodes with higher degrees exhibit greater centrality. Distinct colors represent different modules. Edges denote strong (Spearman’s *ρ* > 0.6) and significant (*P* < 0.01) correlation. (**E**) Pearson correlations between network complexity and stability indices, with significant correlations (*P ＜ 0.05) highlighted. Positive correlations are indicated in blue, while negative correlations are shown in orange. Correlation coefficients were displayed in cells. Correlations with *P* > 0.5 were omitted.

Four networks were constructed to evaluate the bacterial network complexity (Fig. 5A through D). As shown in Table 1, the bacterial co-occurrence networks at different stages of CC displayed distinct patterns based on network topological parameters. Network metrics (nodes, links, and modules) peaked during MS, following a hump-shaped trend, while avgK, GD, and avgCC showed trough-like patterns (HS-MS-PS). Keystone species counts similarly exhibited unimodal variation (Fig. S3A through D). These patterns aligned with microbial diversity dynamics (Fig. 3C and E; Fig. S1E), suggesting enhanced microbial connectivity and network stability during MS satge. Interactions at different soil stages predominantly exhibited positive correlations, with maximum positive correlation in HS stage (75.98%) and minimum in PS stage (58.82%) excluding the SS stage (Fig. 5B and D).

**TABLE 1:** Topological properties of bacterial networks at different stages^*a*^

Communities	SS	HS	MS	PS
No. of OTUs	1784	3586	6757	5045
Total nodes	38	717	1166	647
Total links	70	970	1496	867
No. of modules	9	107	176	96
Modularity	0.46	0.91	0.91	0.92
*R*^2^ of power law	0.66	0.90	0.95	0.92
AvgK	3.68	2.71	2.57	2.68
GD	1.98	10.56	10.19	11.00
HD	1.56	7.09	7.91	7.47
No. of keystones	0	6	11	5
AvgCC	0.40	0.28	0.20	0.27
Con	0.26	0.22	0.38	0.24

^*a*^ AvgK, average degree; GD, average path distance; avgCC, average clustering coefficient; Con, connectedness.

To evaluate stage-specific impacts on bacterial network stability, we simulated species extinction to calculate robustness. Random removal demonstrated the highest robustness in SS stage, with sharp declines through the HS, MS, and PS stages (Fig. S3E). Network vulnerability during MS stage (Fig. S3F), indicating the greatest instability at this stage. Notably, stability metrics showed significant correlations with network complexity. Robustness negatively correlated with complexity indices (links, nodes, HD, GD, and modularity; Pearson’s *r* = −0.95 to −0.99, *P* < 0.05). Vulnerability and robustness were positively correlated with Con, avgK, and avgCC (*r* = 0.95–0.99, *P* < 0.05, Fig. 5E).

### Linking bacterial communities with soil physicochemical properties and enzyme activities

**Fig 6:**
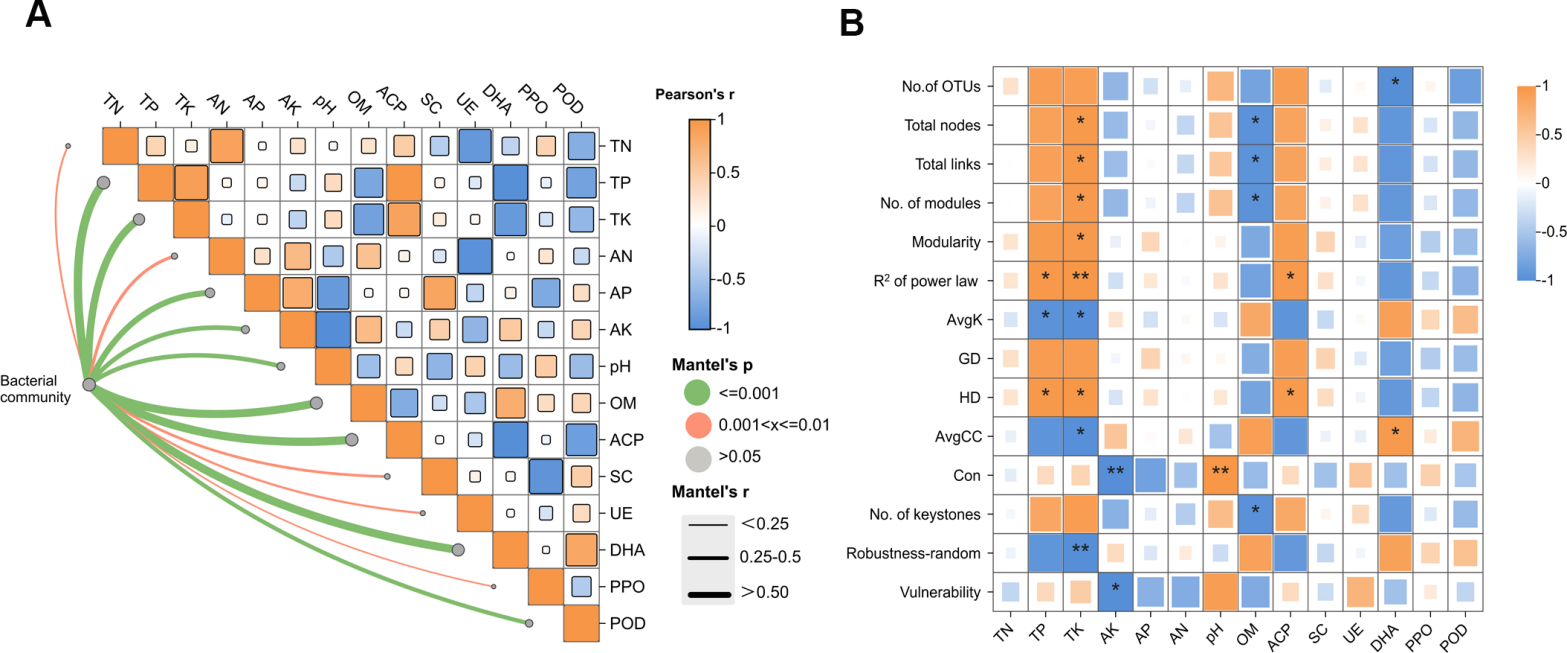
Relationships between bacterial communities, soil physicochemical properties, and enzyme activities. (**A**) Mantel test results show associations between bacterial composition (Bray-Curtis distance) and soil parameters/enzyme activities. Color intensity indicates significance level (*P* values). Thickness corresponds to Mantel’s *R* coefficient magnitude. (**B**) Pearson correlation between network stability and network complexity indices and soil parameters (*P* < 0.05). Significant correlations are marked with asterisks.

Mantel tests revealed significant correlations between bacterial communities and key soil parameters (TP, TK, AP, AK, and OM) as well as enzyme activities (ACP, DHA, and POD) across CC developmental stages (Fig. 6A). Network analysis demonstrated that microbial network stability and complexity were strongly influenced by TP, TK, AK, pH, OM, ACP, and DHA (Fig. 6B). Our findings demonstrated that microbial network characteristics were significantly related to TP, TK, AK, pH, OM, ACP, and DHA. Specifically, TP was positively correlated with the HD in the network. TK was positively correlated with the total number of links, nodes, modules, and HD in the network. pH was positively correlated with Con. TP, TK, and pH were associated with increased network complexity. These patterns aligned with observed stage-specific changes: network complexity peaked during the MS stage (Fig. 1D and E), coinciding with maximum TP, TK, and pH levels but minimal AK and OM content (Fig. 1B and H). Our results demonstrate that CC development dynamically alters soil properties, which in turn govern microbial network structure, with the MS stage showing the most pronounced effects on network complexity.

### Functional profiling of soil microbial communities

**Fig 7:**
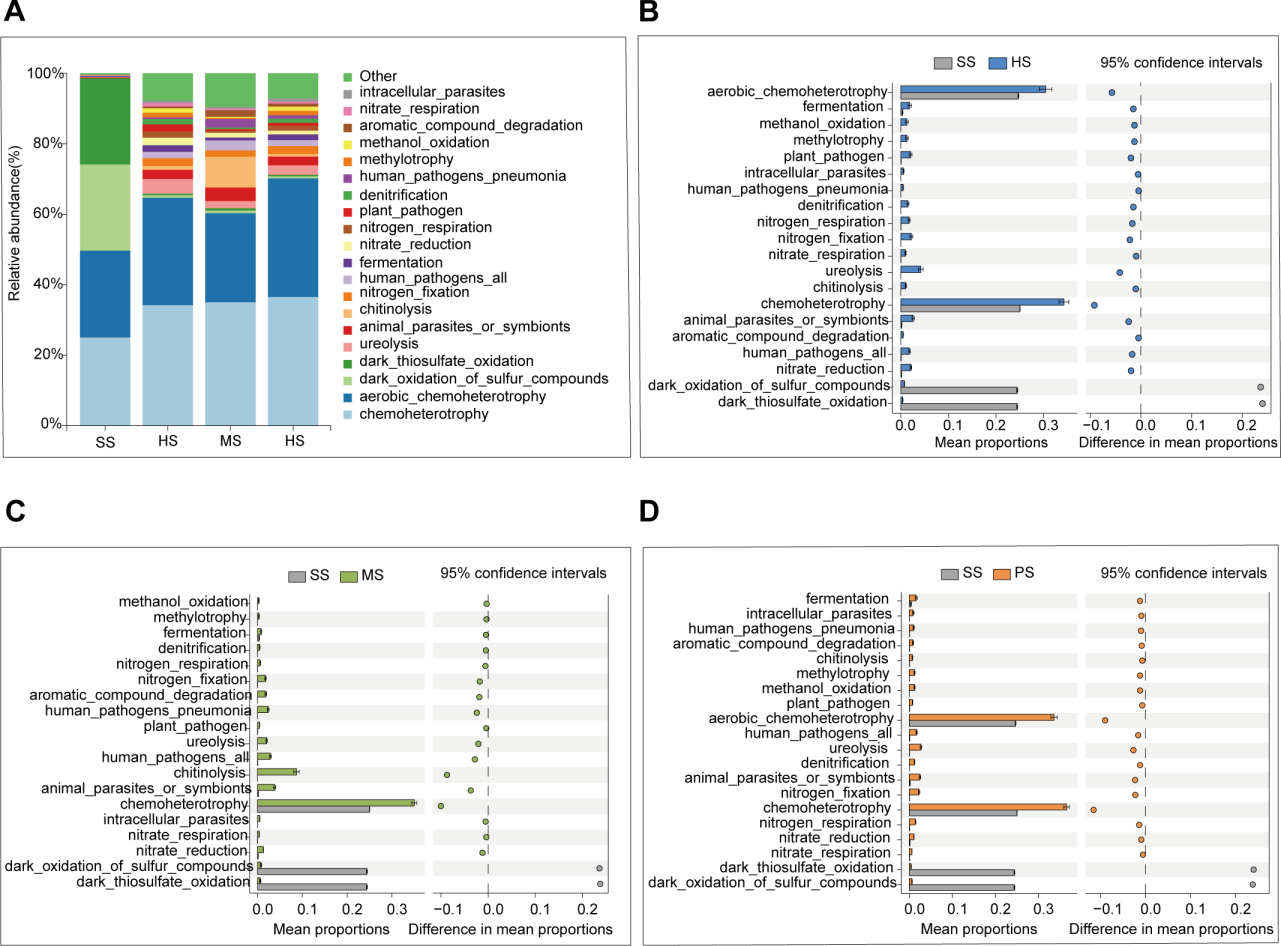
Putative functions of microbial communities via FAPROTAX. (**A**) Relative abundance of predicted bacterial functional at different stages. (B–D) Differential functional abundance (*t*-tests): (**B**) SS vs HS, (**C**) SS vs MS, and (**D**) SS vs PS.

To explore the functional characteristics of soil microorganisms of CC at different stages, the top 20 ecological functions with high abundance were predicted based on the FAPROTAX database. The results revealed chemoheterotrophy and aerobic chemoheterotrophy as the dominant ecological functions across all CC developmental stages (Fig. 7A). The abundance of dark oxidation of sulfur compounds and dark thiosulfate oxidation in SS stage was significantly higher than that of HS, MS, and PS stages (Fig. 7A). These results suggested that CC with both functions were gradually consumed as they grew. Chemoheterotrophy and aerobic chemoheterotrophy in the HS and PS stages were significantly higher than in the SS stage (Fig. 7B through D). Still, they were the most abundant in the PS stage (Fig. S4), indicating that microorganisms with these functions gradually enriched after the formation of CC. Notably, chemoheterotrophy and chitinolysis in the MS stage were significantly higher than in the SS stage (Fig. 7C), and MS accounts for the highest proportion in the four stages (Fig. S4). The results showed that the microorganisms with this function were significantly enriched in the MS stage (Fig. S4).

## DISCUSSION

Our study revealed dynamic changes in soil physicochemical properties and enzyme activities during CC formation. Specifically, pH, OM, TP, TK, AP, AK, ACP, and DHA fluctuated sharply, which reflected the complex process of nutrient absorption, transformation, and release during CC cultivation.

The soil pH dynamics during CC cultivation exhibited a characteristic shift from acidic conditions (SS and HS stages) to alkaline (MS stage) (Fig. 1A), reflecting fungal-mediated pH modification through metabolic activities. In the HS stage, the acidic soil environment promoted fungal colonization and invasion (27, 28). However, following successful infection in the MS stage, the release of organic acids or alkalis during fungal metabolism adjusted the soil pH, potentially aiding immune escape, tissue destruction, or stimulating reproduction (29). This process may destroy the host larvae’s immune system, accelerate CC growth, and thus promote its formation. This alkaline shift during MS aligns with established optimal pH ranges for mycelial growth (30, 31). OM decreased by 30.61% in the HS stage and 45.45% in the MS stage (Fig. 1B), reflecting larval nutrient consumption and decomposition processes. In addition, TP and TK increased during the HS, MS, and PS stages, with a notable 76.04% increase in TP in the MS stage, while AP and AK declined sharply (Fig. 1). This indicates that the fungi absorb nutrients at this stage, highlighting the need for P and K for mycelium growth during CC formation. These observations are consistent with previous studies emphasizing the essential role of P and K in fungal development (32, 33).

Moreover, dynamic variations in soil enzyme activities (particularly ACP and DHA; Fig. 2) revealed their critical roles in CC formation. Studies have shown that soil enzymes enhance soil properties by accelerating nutrient transformation and cycling. ACP activity paralleled the increasing TP content during CC development, consistent with its known function in regulating P availability (34, 35). This correlation suggests ACP facilitates CC formation by mobilizing soil P reserves. DHA, a robust indicator of microbial metabolic activity (36), reached its lowest level during the MS stage corresponding to maximal OM depletion and peak fungal growth. This inverse relationship demonstrates intensive nutrient consumption during mycelial expansion, confirming that soil biochemical processes may influence host larval development and CC morphogenesis, particularly during this critical transitional stage.

Our study revealed dynamic microbial community shifts during CC formation, with particularly pronounced changes during the MS stage. Microbial diversity analysis demonstrated significant fluctuations in bacterial OTUs across developmental stages, with the MS stage exhibiting the highest number of unique OTUs, peak species richness, and maximal community diversity (Fig. 3 and 4, and Fig. S2). These findings suggested that microbial alterations during the MS stage were crucial for CC formation. This observation aligns with the concept that microbial communities become more diverse as soil undergoes ecological succession or development (37, 38). Further analysis of microbial phyla and genera showed an increased relative abundance of Proteobacteria, Verrucomicrobia, and Firmicutes in the MS stage. These trends align with patterns observed in soil development and OM decomposition (39, 40). That is to say, the microorganisms of these phyla may participate in the formation of CC by affecting soil development and OM decomposition. It is worth noting that Proteobacteria and Verrucomicrobiota exhibit the higher relative abundance. Previous studies have emphasized the universality of these bacteria in soils with high OM content and rapid growth (41, 42). Recent findings indicate that changes in host-associated microbial communities, particularly the presence of Firmicutes, can inhibit bacterial wilt in tomatoes (43). The observed increase in Firmicutes in our study may have played a significant role in disease suppression, thereby promoting the formation of CC. At the genus level, the relative abundance of *Dyella*, *Chitinophaga*, *Flavobacterium*, and *Pseudomonas* tended to increase. Previous studies have shown that *Dyella* can promote OM decomposition and increase infection rates (44, 45). *Chitinophaga* plays a key role in carbohydrate processing in soils, while *Flavobacterium* aids in P uptake and inhibits pathogens (46). *Pseudomonas* has been shown to stimulate mycelium growth, promote primordium formation, induce fruiting body development, and increase mushroom yield (47, 48). Additionally, *Pseudomonas* supports nutrient absorption by the *Cordyceps militaris* host (49), suggesting that *Pseudomonas* likely facilitated CC formation in our study.

In addition to changes in microbial diversity, the most significant alterations in the complexity and stability of the microbial network occurred in the MS stage. Microbial network analysis reveals random interactions among microorganisms, reflects the structure of complex microbial communities, and provides insights beyond sample-level comparisons (50). In soil ecosystems, microbial network analysis further elucidated microbial community dynamics (51). Our results showed that CC formation increased the network’s complexity while reducing its stability (Fig. 5 and Fig. S3). In terms of complexity, the network’s number of nodes and modules increased sharply in the MS stage, indicating that microbial interaction was more tightly interconnected at this stage. Previous studies have demonstrated that alterations in microbial network complexity affect soil ecological functions, thereby influencing species growth (52, 53), findings consistent with our observations. Moreover, research suggests that network complexity is positively correlated with bacterial diversity, potentially explaining the increased network complexity observed during the MS stage, as more diverse microbial groups enhance the likelihood of complex interaction (50). Stability analysis revealed that the MS stage exhibited the lowest network robustness and highest vulnerability, indicating that the microbial network was more susceptible to disturbances. We speculate that decreased stability may result from rapid shifts in microbial community structure and soil conditions during the mummification process. At MS stage, strong microbial interactions likely exacerbate the formation of CC. Thus, while elevated network complexity reflects intensified microbial interactions, it concomitantly highlights potential instability. This aligns with the theoretical model, which posits that higher complexity may lead to ecosystem instability (26). Consequently, increased network complexity and decreased stability were observed during the MS stage. These changes underscore intensive microbial interference occurring at this critical point in CC formation.

Furthermore, according to FAPROTAX analysis, the chitinolytic activity in MS-stage soil was significantly higher compared to the HS and PS stages (Fig. 7). This is likely due to the chitin in the larvae’s exoskeleton. Chitin-degrading bacteria facilitating the invasion of host larvae at this stage, weakening their immune system and promoting microbial colonization. Previous studies suggested that bacteria such as *Pseudomonas* and *Flavobacterium* produce chitinase and participate in the invasion process (54, 55). These bacteria secrete chitinase, which is involved in the invasion of host larvae during the MS stage of CC formation. FAPROTAX is effective for predicting the function of bacteria and can provide insights into various microbial activities. However, its ability to infer metabolic phenotype relies solely on cultured microbial representatives. As more microorganisms are cultured, some of the predicted functions may be inaccurate (56). Additionally, FAPROTAX relies on generalized functional profiles of taxa, fails to account for context-dependent functional variations, and cannot capture more complex or specialized functions (57). Further studies are needed to explore the specific functions of these microorganisms.

The formation of CC occurs entirely within the soil, which plays a crucial role in its growth and development. Soil physicochemical properties, enzyme activities, and microbial communities collectively drive CC formation, while the presence of CC, in turn, alters soil properties and microbial interactions, creating a dynamic feedback loop. In this study, the MS stage was identified as the critical stage in CC formation. At this stage, changes in soil properties coincided with increased microbial richness and diversity. This led to a more complex but unstable network, which further disrupted microbial function and ultimately facilitated CC formation. Future studies will investigate the role of key microbial communities across different soil stages. Understanding how specific microbes affect soil processes and CC development will guide targeted interventions aimed at optimizing soil health and enhancing CC production.

### Conclusion

Our study elucidates the dynamic interplay between soil properties, enzymatic activities, and microbial communities that influence the growth and development processes of CC. Firstly, soil physicochemical properties showed significant fluctuations throughout the CC formation stages. Notably, pronounced nutrient depletion was observed during the larval feeding and mummification phases. Secondly, microbial communities underwent substantial shifts during the CC formation process. Microbial network analysis revealed the MS stage exhibited the highest network complexity yet also the greatest vulnerability. This indicates microbial communities at this stage are more susceptible to perturbations. Additionally, the predicted functional properties of these microbial communities indicated a shift in ecological functions, with chemoheterotrophy and chitinolysis becoming more prominent during the MS stage. This reflects microbial adaptations that support CC formation. Our study lays the foundation for further elucidation of the molecular mechanisms behind microbial interactions in soil and their role in optimizing CC production in the future. Future research could focus on the functional roles of specific microbial taxa, the impact of soil amendments on microbial network stability, and the long-term effects of CC cultivation on soil health and ecosystem services.

## Data Availability

Sequencing data are available in the NCBI BioProject database (BioProjectID: PRJNA1241115).

## SUPPLEMENTAL MATERIAL

Supplemental material spectrum.03277-24-s0001.docx Fig. S1 to S4; Table S1.

ASM does not own the copyrights to Supplemental Material that may be linked to, or accessed through, an article. The authors have granted ASM a non-exclusive, world-wide license to publish the Supplemental Material files. Please contact the corresponding author directly for reuse.
