# Supplementary material for: The interplay between the formation of Chinese cordyceps and the characteristics of soil properties and microbial network
Source: Microbiol Spectr. 2025 May 30;13(7):e03277-24. doi: 10.1128/spectrum.03277-24 (PMC12211017; doi:10.1128/spectrum.03277-24)
Supplement: Supplemental material — Fig. S1 to S4; Table S1. [file spectrum.03277-24-s0001.docx]

**The interplay between formation of Chinese cordyceps and the characteristics of soil properties and microbial network**

Qinghe Wang ^1^, Yapei Wang ^1^, Ting Li ^1^, Xiuwen Bao ^1^, Liying He ^1^, Lin Liu ^1^, Sijing Liu ^2^, Jing Bai ^2^, Han Zhang ^1^, Shuqi Niu ^2∗^, Jinlin Guo ^1∗^

^1^School of Pharmacy, Chengdu University of Traditional Chinese Medicine, Chengdu 611137, China

^2^College of Medical Technology, Chengdu University of Traditional Chinese Medicine, Chengdu 611137, China

^∗^Address correspondence to Shuqi Niu and Jinlin Guo, [niushuqi@cdutcm.edu.cn](mailto:niushuqi@cdutcm.edu.cn); [guo596@cdutcm.edu.cn](mailto:guo596@cdutcm.edu.cn)

**Supplementary Figures**


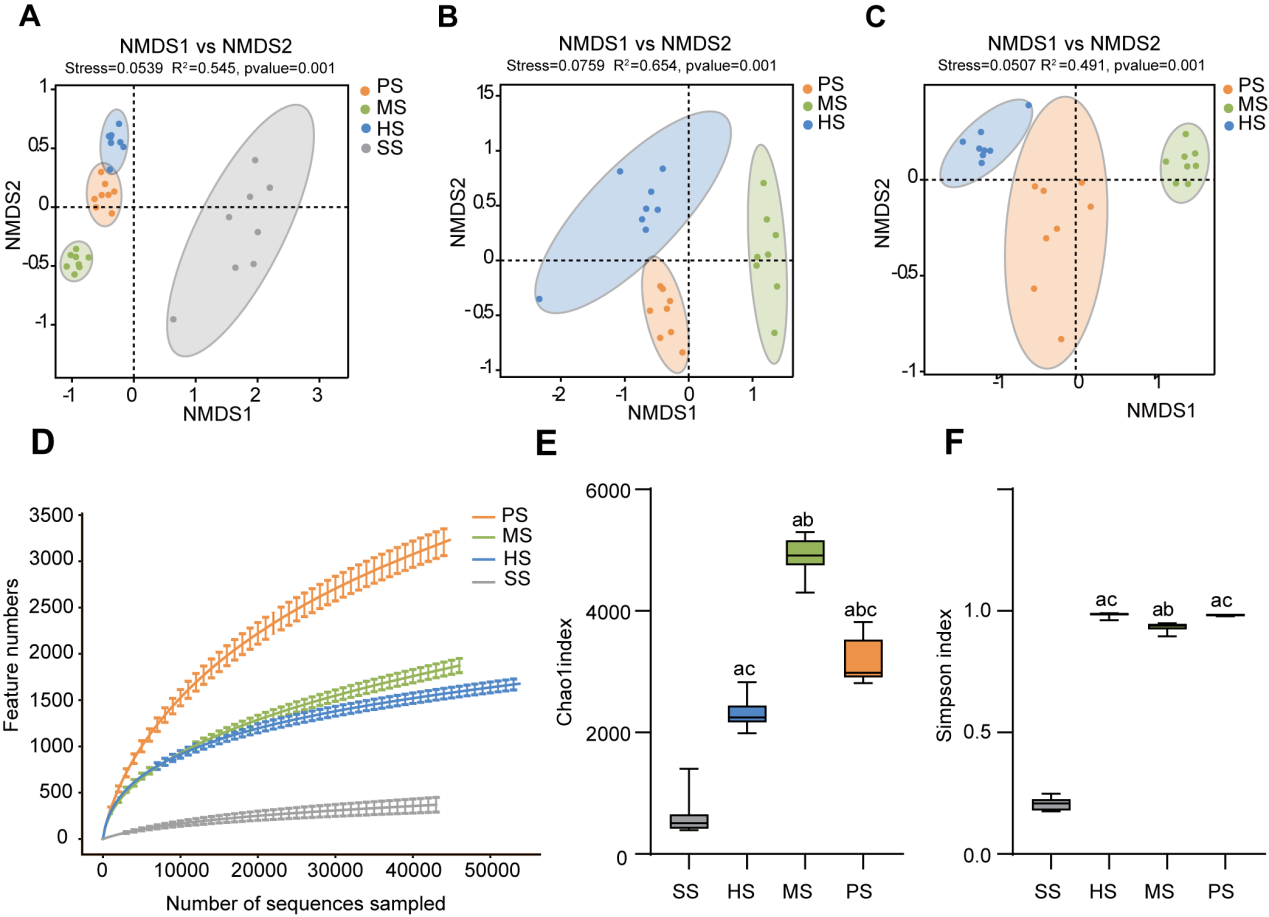


**FIG S1** Microbial community diversity in the soil at different stages of CC. (A) NMDS analysis of the microbial community in SS, HS, MS and PS stages using unweighted UniFrac distance metric. (B) NMDS analysis of microbial community in HS, MS and PS stages using weighted UniFrac distance metric. (C) NMDS analysis of the microbial community in HS, MS and PS stages using unweighted UniFrac distance metric. (D) Rarefaction curves. (E) Chao1 index. (F) Simpson index. **Note:** Lowercase letters indicate significant differences (*P* < 0.05): a vs SS; b vs HS; and c vs MS.


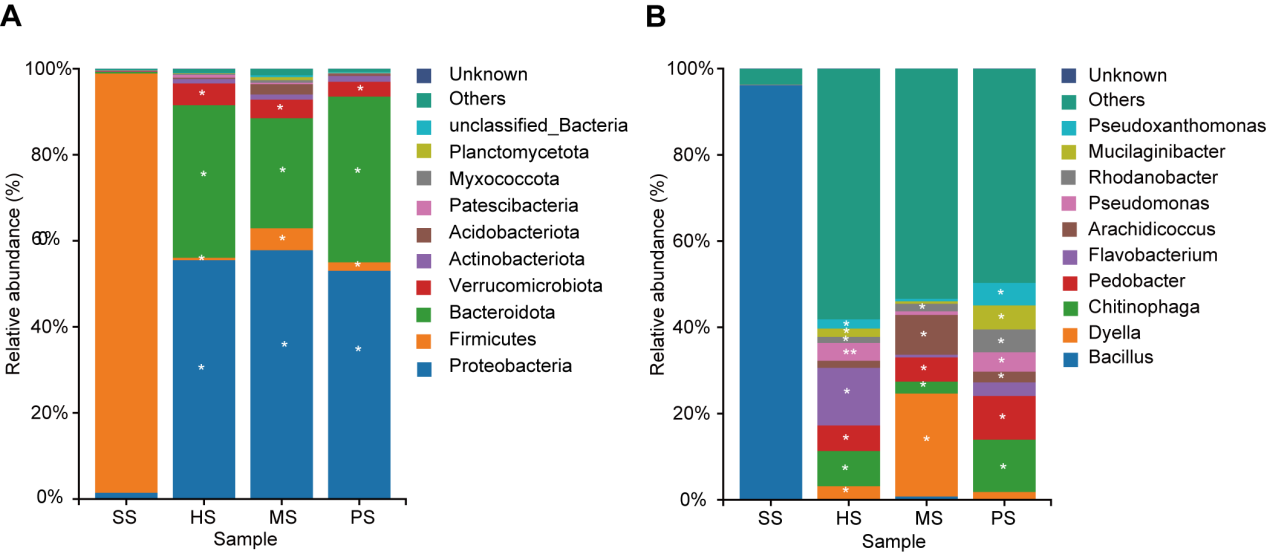


**FIG S2** Relative abundance of the top 10 most abundant orders in control SS and HS, MS, and PS stages. (A) Phyla level. (B) Genus level. **Note:** Different significance levels between SS and HS, MS stages, and PS are marked with asterisks (**P* < 0.05). Low abundance orders (*P*＞0.05) with insignificant differences aren’t marked with asterisks.

**
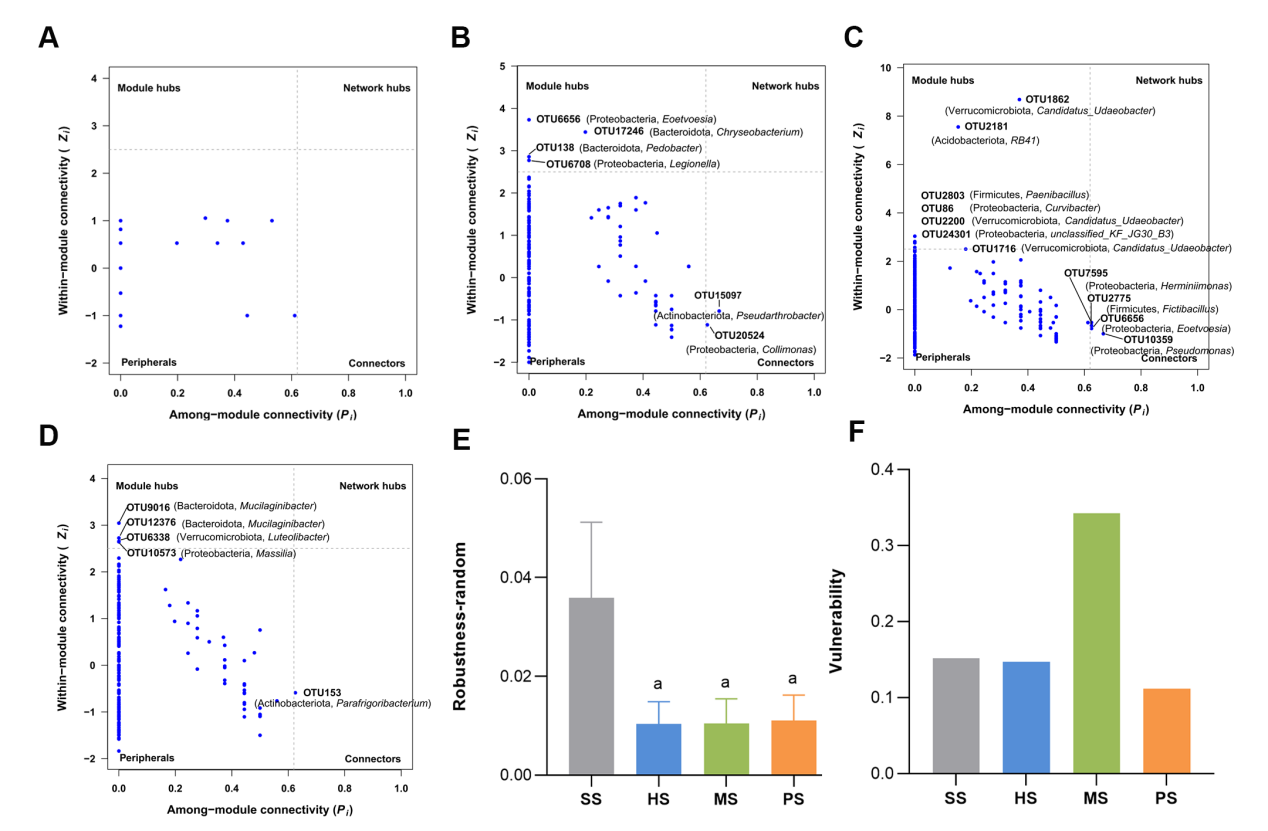
FIG S3** Network complexity and stability analysis for the bacterial community. (A) *Zi*-*Pi* plot for different groups. *Zi*-*Pi* plot illustrates the distribution of nodes based on their topological roles in aggregate-related networks, with module hubs and connectors are labeled with OTU numbers and corresponding bacteria. (B) Robustness is defined as the proportion of taxa that remain after randomly removing nodes from the networks. Error bars represent the standard deviation from 100 simulation repetitions. (C) Network vulnerability is quantified by the maximum node vulnerability in each network.


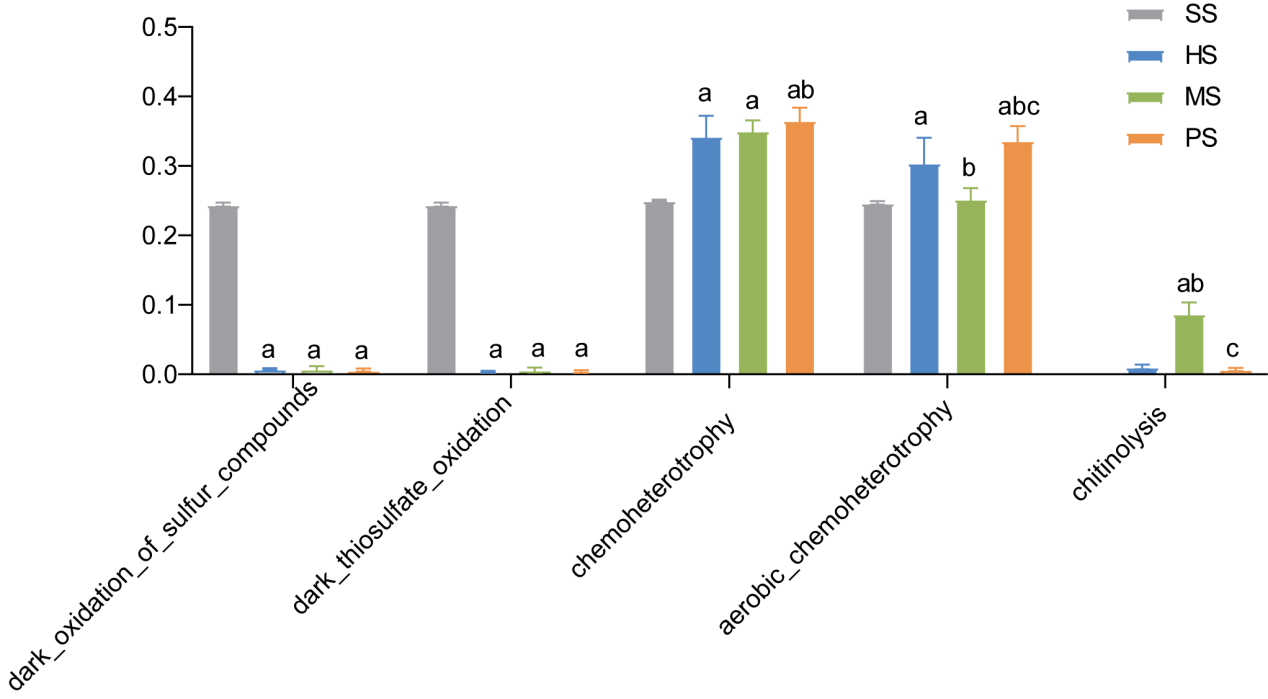


**FIG S4** Comparison of relative abundance of five bacterial functions with the most significant differences predicted using FAPROTAX. **Note:** a indicates a significant difference with SS (*P* < 0.05); b suggests that there is a substantial difference with HS (*P* < 0.05); c represents a considerable difference with MS (*P* < 0.05 ).

**Supplementary Table**

**Table S1.** Compared with SS stage, the classification information of the enriched common OTUs between HS, MS, and PS stages

| **ID** | **Phylum** | **Class** | **Order** | **Family** | **Genus** |
| --- | --- | --- | --- | --- | --- |
| OTU101 | Bacteroidota | Bacteroidia | Chitinophagales | Chitinophagaceae | *Chitinophaga* |
| OTU10478 | Proteobacteria | Gammaproteobacteria | Burkholderiales | Comamonadaceae | *Rhizobacter* |
| OTU12471 | Bacteroidota | Bacteroidia | Sphingobacteriales | Sphingobacteriaceae | *Pedobacter* |
| OTU12779 | Actinobacteriota | Actinobacteria | Micrococcales | Microbacteriaceae | *Galbitalea* |
| OTU128 | Bacteroidota | Bacteroidia | Sphingobacteriales | Sphingobacteriaceae | *Pedobacter* |
| OTU12987 | Proteobacteria | Gammaproteobacteria | Burkholderiales | Alcaligenaceae | *Verticiella* |
| OTU13089 | Actinobacteriota | Actinobacteria | Micrococcales | Microbacteriaceae | *Microbacterium* |
| OTU13416 | Proteobacteria | Gammaproteobacteria | Burkholderiales | Comamonadaceae | *Ramlibacter* |
| OTU137 | Bacteroidota | Bacteroidia | Sphingobacteriales | Sphingobacteriaceae | *Pedobacter* |
| OTU14096 | Actinobacteriota | Actinobacteria | Micrococcales | Microbacteriaceae | *Leifsonia* |
| OTU16 | Proteobacteria | Gammaproteobacteria | Xanthomonadales | Rhodanobacteraceae | *Dyella* |
| OTU16044 | Proteobacteria | Gammaproteobacteria | Pseudomonadales | Pseudomonadaceae | *Pseudomonas* |
| OTU1696 | Firmicutes | Bacilli | Lactobacillales | Lactobacillaceae | *Leuconostoc* |
| OTU1742 | Firmicutes | Bacilli | Paenibacillales | Paenibacillaceae | *Paenibacillus* |
